# Accidental Electric Shocks

**Published:** 1902-04-05

**Authors:** 


### Hospital Clinics and Medical Progress.

### ACCIDENTAL ELECTRIC SHOCKS.

In view of the large extension which is taking place in the use of electricity as a motive force on railways and tramways and the recent increase in the voltage of the current supplied for lighting and other purposes much interest attaches to the remarks made by Mr. A. P. Trotter in a paper read before a recent meeting of the Institutions of Electrical Engineers on the subject of "Electric Shocks at 500 Volts." He says that a pressure of 500 volts has become well established as the standard for electric traction, and that conditions of commercial standardisation seem to set the working limit at about 600 volts, so that no further extension of pressure is likely to be needed. Undoubtedly people have been killed by currents of 500 and even of 200 volts, but, as Mr. Trotter points out, for this result to occur peculiar combinations of circumstances must arise which are not likely to be frequently met with, for, when a person receives a shock from a live wire or from coming in contact with the third rail on an electric railway the effect upon him depends not upon the voltage in the wire, but upon the amount of current which is able to pass through his body, which is strictly limited by the resistance offered by his clothes and tissues and by the size of the contact surface.

The pain of a contact depends upon the density of the current rather than its amount. In medical applications a current of more than 20 milliampères is seldom used. It is evident then that the amount of current required to cause great pain and to do great injury is very small, and that even with what are considered low voltages disastrous consequences would follow contact with "live" wires were it not for the fact that under ordinary circumstances we are protected by the resistance offered by our skin and our clothing to the passage of anything more than very small currents. To show how great is this protection Mr. Trotter says that the resistance from finger-tip to finger-tip on dry metal, with currents under 100 volts is about 20,000 ohms. At higher pressures it decreases a little. The resistance is almost all at the skin, the difference between the resistance from finger to finger on one hand and between one finger of each hand being inappreciable. The resistance from hand to hand when grasping two pieces of trolley wire is about 5,000 ohms, but varies a good deal, probably according to the dryness of the skin. Mr. Trotter has found it as high as 14,000 ohms. From boot to boot—the boots being dry and without nails—it varies from 45,000 to more than 200,000. Boots worn into holes, however, and wetted by walking on wet pavements gave only 13,000. Thus, unless a contact is large, or the skin and clothes are wet, there is generally ample resistance to afford protection. The mere touching of dry metal at 100 volts, finger to finger, gives hardly any sensation. At 200 volts a light touch gives an unpleasant prick; but even with a firm contact the current passing through is only about 12 to 18 milliampères, which most people can bear without considerable pain, especially if the contact be made and broken gradually. To grasp with bare hands two pieces of metal at 500 volts would give a very painful shock. A light and quick touch is no worse than the shock from a half-pint Leyden jar, but a bad 500-volt shock burns the skin. To show further how great is the resistance offered by ordinary conditions to the passage of dangerous currents, Mr. Trotter relates that during his inspection of the last extension of the City and South London Railway, he stood on the rails in wetted boots and sat on the live conductor and slapped the running rails with his bare hands. But if the skin resistance be reduced by moisture, especially if salt or chemicals be present, and if the contact be large and prolonged 100 volts may be fatal. This is the great thing to recollect, that safety depends upon the dryness of the skin and the clothes and upon the small area of the contact. In the well-known Liverpool catastrophe the sufferers were entangled in the fallen telephone wires, they were thrown down into the salt slush, and a long time elapsed before the current was cut off. The contacts were thus large, wet and prolonged.

